# Regarding: TACE Versus TARE for Patients With Hepatocellular Carcinoma: Overall and Individual Patient‐Level Meta‐Analysis

**DOI:** 10.1002/cam4.70683

**Published:** 2025-02-13

**Authors:** Xu Feng, Zheng‐Rong Shi

**Affiliations:** ^1^ Department of Hepatobiliary Surgery The First Affiliated Hospital of Chongqing Medical University Chongqing China

Dear Editors,

We read with great interest the article by Brown et al. [1], which included 17 studies with one randomized trial, 4 prospective studies, and 12 retrospective studies. We are very grateful to the authors for their contribution to the comparison of the efficacy of transarterial radioembolization (TARE) and transarterial chemoembolization (TACE) of hepatocellular carcinoma. However, several methodological issues in the article are worthy of comment.

With regard to TACE, the paper included eight studies using conventional TACE (cTACE), five studies using drug‐eluting bead transarterial chemoembolization (DEB‐TACE), three studies using both cTACE and DEB‐TACE, and one study that used TACE with degradable starch microspheres (DSM‐TACE). Although cTACE, DEB‐TACE, and DSM‐TACE all belong to TACE, there are still some differences in the treatment methods. As we know, there is a significant difference in efficacy between cTACE and DEB‐TACE [2, 3]. Although one study showed that regarding local tumor response and overall survival, the results of DSM‐TACE were similar to those of cTACE [4], research on DSM‐TACE versus cTACE with DEB‐TACE is still lacking. Therefore, we did not think that this meta‐analysis should include both cTACE, DEB‐TACE, and DSM‐TACE, which would introduce significant bias into the studies. Meanwhile, we did not consider it appropriate to include studies combining cTACE with DEB‐TACE. Therefore, these may be one of the important sources of apparent heterogeneity in the paper. We guessed it would be a better option to compare TARE versus cTACE alone and TARE versus DEB‐TACE in the paper or subgroup analysis.

In addition, we are somewhat confused about the article by Biederman et al. [5]. In the meta‐analysis, the study was divided into the DEB‐TACE group, but after we repeatedly read the original text, we did not clearly figure out the type of TACE in the research.

In conclusion, we appreciate the authors' efforts in the treatment of hepatocellular carcinoma. However, improvements in the article would further support and solidify the conclusions made in this study.

## Author Contributions

**Xu Feng:** writing – original draft (equal). **Zheng‐Rong Shi:** writing – review and editing (equal).

## Conflicts of Interest

The authors declare no conflicts of interest.

## Data Availability

The authors have nothing to report.

## References

[1] A. M. Brown , I. Kassab , M. Massani , et al., “TACE Versus TARE for Patients With Hepatocellular Carcinoma: Overall and Individual Patient Level Meta Analysis,” Cancer Medicine 12 (2023): 2590–2599, 10.1002/cam4.5125. 35943116 PMC9939158

[2] J. H. Zou , L. Zhang , Z. G. Ren , and S. L. Ye , “Efficacy and Safety of cTACE Versus DEB‐TACE in Patients With Hepatocellular Carcinoma: A Meta‐Analysis,” Journal of Digestive Diseases 17 (2016): 510–517, 10.1111/1751-2980.12380. 27384075

[3] N. Peng , L. Mao , Y. Tao , K. Xiao , G. Yuan , and S. He , “Callispheres® Drug‐Eluting Beads Transarterial Chemoembolization Might Be an Efficient and Safety Down‐Staging Therapy in Unresectable Liver Cancer Patients,” World Journal of Surgical Oncology 20 (2022): 254, 10.1186/s12957-022-02717-9. 35941634 PMC9361543

[4] C. Niessen , E. Unterpaintner , H. Goessmann , et al., “Degradable Starch Microspheres Versus Ethiodol and Doxorubicin in Transarterial Chemoembolization of Hepatocellular Carcinoma,” Journal of Vascular and Interventional Radiology 25 (2014): 240–247, 10.1016/j.jvir.2013.10.007. 24291001

[5] D. M. Biederman , J. J. Titano , R. A. Korff , et al., “Radiation Segmentectomy Versus Selective Chemoembolization in the Treatment of Early‐Stage Hepatocellular Carcinoma,” Journal of Vascular and Interventional Radiology 29 (2018): 30–37, 10.1016/j.jvir.2017.08.026. 29169782

